# Vision on gyrate atrophy: why treat the eye?

**DOI:** 10.1038/s44321-023-00001-1

**Published:** 2023-12-14

**Authors:** Arthur A Bergen, Mark JN Buijs, Anneloor LMA ten Asbroek, Berith M Balfoort, Camiel JF Boon, The Dutch GACR “Bird’s Eye View” Consortium, Roselie RMH Diederen, Sacha Ferdinandusse, Elise A Ferreira, Patrick Schultink, Corrie Timmer, Frédéric M Vaz, Joost Verhaagen, Margreet AEM Wagenmakers, Hans R Waterham, Frits Wijburg, Marion M Brands, Ronald JA Wanders, Clara DM van Karnebeek, Riekelt H Houtkooper

**Affiliations:** 1 Department of Human Genetics, Section Ophthalmogenetics, Amsterdam UMC, University of Amsterdam, 1105 AZ Amsterdam, the Netherlands; 2 Department of Ophthalmology, Amsterdam UMC, University of Amsterdam, 1105 AZ Amsterdam, the Netherlands; 3 Emma Center for Personalized Medicine, Amsterdam UMC, Amsterdam, the Netherlands; 4 Department of Paediatrics, Emma Children’s Hospital, Amsterdam UMC, University of Amsterdam, 1105 AZ Amsterdam, the Netherlands; 5 Laboratory Genetic Metabolic Diseases, Amsterdam Gastroenterology, Endocrinology, and Metabolism, Amsterdam UMC, University of Amsterdam, 1105 AZ Amsterdam, the Netherlands; 6 Department of Ophthalmology, Leiden UMC, Leiden, the Netherlands; 7 United for Metabolic Diseases, Amsterdam, The Netherlands; 8 VKS: Dutch Patient Organization for (Inherited) Metabolic Diseases, Zwolle, Netherlands; 9 Departments of Dietetics and Nutritional Science and Endocrinology and Metabolism, Amsterdam University Medical Centers, Amsterdam, the Netherlands; 10 Netherlands Institute for Neuroscience, A Research Institute of the Royal Netherlands Academy of Sciences, Meibergdreef 47, 1105BA Amsterdam, The Netherlands; 11 Department of Internal Medicine, Centre for Lysosomal and Metabolic Diseases, Erasmus MC, University Medical Centre Rotterdam, Rotterdam, the Netherlands

**Keywords:** Genetics, Gene Therapy & Genetic Disease

## Abstract

In the April issue of this Journal, Boffa and coworkers put forward a new therapeutic approach for Gyrate Atrophy of the Choroid and Retina (GACR; OMIM 258870) (Boffa et al, 2023). The authors propose to apply gene therapy to the liver for GACR, a metabolic disease primarily affecting eyesight due to retinal degeneration. Their vision is enthusiastically supported by a *News and Views* comment in the same issue (Seker Yilmaz and Gissen, 2023). However, based on disease pathology, patient’s needs, ethical considerations, therapeutic developmental time lines, and current state of the art of gene therapy for liver and eye, we have a different view on this issue: We argue below that local treatment of the eye is the preferred option for GACR.

## Abstract (web summary)

In this Correspondence, R Houtkooper and colleagues argue that local treatment of the eye is the preferred option for treatment of gyrate atrophy of the choroid and retina.

## Graphical abstract

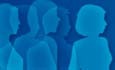

GACR is a rare autosomal recessive disorder of ornithine metabolism. Clinically, GACR is characterized by progressive chorioretinal atrophy. Patients present in early childhood with complaints of myopia and nyctalopia. Eventually, the disease leads to severe visual impairment and functional blindness by the fourth–fifth decade of life. Other (neurological) symptoms have been reported, but not consistently, and are often not of clinical significance (Balfoort et al, 2021). Patients present with accumulation of ornithine in body fluids, such as plasma, urine, cerebrospinal fluid, and aqueous humor. Why the retina is primarily affected is not entirely clear.

GACR is caused by loss-of-function mutations in *OAT*. This gene encodes ornithine-δ-aminotransferase (OAT), which catalyzes the reversible reaction of ornithine and α-ketoglutarate to glutamate and glutamate-5-semialdehyde (GSA). The latter exists in a biochemical equilibrium with δ1-pyrroline‐5‐carboxylate (P5C), which can be converted to proline (Fig. 1). In terms of pathophysiology, it is not certain whether the disease is caused by high ornithine levels, or if absence of downstream metabolites is causing damage to cells. Indeed, clinical and experimental evidence suggest that high levels of ornithine can damage retinal pigment epithelial (RPE) cells directly (Wang et al, 2000). At the same time, it should be noted that the majority of patients suffering from hyperornithinemia–hyperammonemia–homocitrullinuria syndrome (HHH syndrome; OMIM 238970), caused by mutations in the mitochondrial ornithine transporter *SLC25A15*, also have markedly elevated ornithine plasma levels, but generally do not develop ophthalmic symptoms (Martinelli et al, 2015). It is also not exactly clear whether GACR tissue damage is invoked systemically, locally, or by a combination of both. In this context, it is important to note that, although expression of *OAT* in the liver is high, this gene is also significantly expressed in multiple other cell types in the human body, including the RPE and photoreceptors (Wang et al, 2000). Finally, ornithine production, secretion, local accumulation as well as transport between cells, tissues and body compartments is not fully understood. Indeed, a range of ornithine transporters exists that can potentially influence or regulate systemic and local ornithine levels.

**Figure 1:**
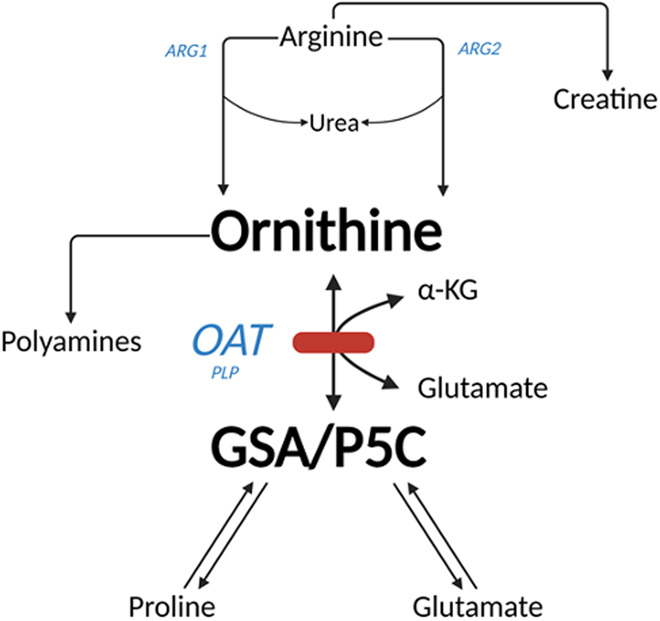
Metabolic pathway of ornithine metabolism. The formation of ornithine from arginine is catalyzed by ARG1 (located in cytoplasm) or ARG2 (located in mitochondria). Ornithine and α-ketoglutarate are converted to glutamate and GSA in a reaction catalyzed by OAT. GSA is in equilibrium with P5C from which proline can be generated. ARG1 Arginase 1, ARG2 Arginase 2, OAT ornithine-δ-aminotransferase, GSA glutamate-5-semialdehyde, P5C δ1-pyrroline‐5‐carboxylate. Created with BioRender.com.

Current GACR treatment focuses on lowering plasma ornithine levels by following a protein-restricted diet, which lowers ornithine production and accumulation. Alternatively, some patients undergo supplementation with pyridoxine (vitamin B6), which supposedly increases residual OAT activity. Compliance to these diets is challenging, and even when patients strictly adhere to the dietary guidelines vision loss is not prevented, only slowed down (Balfoort et al, 2021). New treatments for GACR are being investigated. These interventions include (a) nutritional supplements, (b) repurposed drugs, (c) genetic therapy to prevent short-term (RNA therapy) or long-term (AAV therapy) progression of the disease and (d) cell replacement therapy. Each treatment has its own *pro and cons*, including patient needs and ethical considerations, timeline of development and application, current scientific and technical state of-the art, as well as safety and efficacy.

## Patient needs

Obviously, when designing or applying a potential new therapy, patients’ unmet needs and ethical considerations come first. What does the community, consisting of patients, physicians and scientists think about the proposed therapy? What is the big health advantage and social or practical burden for the patient? What are the alternatives, benefits and risks? For GACR, patients and their relatives consistently report the developing visual handicap as the most devastating feature of the disease (Schultink, 2023). Indeed, in recent surveys, patients with (genetic) eye disorders considered their loss of quality of life comparable to that of Alzheimer’s disease or certain forms of cancer (Scott et al, 2016). In this respect, it is of utmost importance to treat the eye in GACR.

## Ethical considerations

As discussed above, there is currently no effective treatment for GACR. Dietary protein restriction aimed at lowering systemic levels of ornithine at most slows progression of this disease, but does not prevent or halt it. Most other forms of treatment under development will likely take years before they can be translated to patients. Like dietary amino acid restriction, future gene therapy to replace the defective *OAT* in the liver has the purpose to lower systemic ornithine levels, and experimental evidence indicates that it may (partly) prevent clinical symptoms. However, this poses a number of ethical dilemmas: assuming the same treatment effect, should patients (want to) be treated with a life-long diet that is difficult to adhere to, or should they (want to) take a potentially low efficacy liver treatment at early age, with possible systemic toxicity risks? Is it ethical to treat an organ that does not show clinical signs of GACR pathology itself (the liver) with potential risks involved, in order to treat diseased organs elsewhere in the body (the eye)? Following the same line of reasoning, as alternative strategy, one could even consider overexpressing one of nature’s ornithine pumps in the kidney, to secrete excess ornithine from the blood to the urine. It must be acknowledged that whatever the therapeutic intervention, there is value in lowering systemic ornithine levels, if indeed it potentially delays vision loss. However, with this type of treatment, patients will still be confronted with blindness later in life. Thus, if we are able, a priori, to choose and design a new effective treatment, targeting the eye locally with the long-term benefit to maintain good vision seems highly preferable. Additionally, local treatment will “only” result in local potential AAV adverse toxicity effects in the semi-closed compartment of the eye, while systemic toxicity may be life-threatening. Of note, if we would be able to successfully treat the eye of people living with GACR locally at young age, it is not impossible that otherwise degenerative extra-ocular subclinical features may pop up in the ageing GACR patient. Also, there may be still some post-treatment local damage outside the retinal AAV injection area. In our view, it is therefore advisable to explore the future treatment options in combination; e.g. local gene therapy with mild dietary restrictions and/or (local/systemic) protective supplements, to prevent this.

## Developmental timeline of therapies

As GACR is a progressive disease starting at early age, early detection and intervention are essential for preventive treatment strategies. The developmental timeline for any effective treatment is of utmost importance for patients. This timeline does not only include (pre-)clinical research and clinical trials but also more general issues, like the presence of an academic platform or company that makes the treatment available to patients, and the affordability of the proposed treatment.

As mentioned above, potential treatments can be divided in dietary supplements, (repurposed) drugs, gene therapy, and cell therapy. The possibility to create induced pluripotent stem cell-derived human (GACR) models-in-a-dish, in combination with selective use of small animal models, provides an excellent new opportunity to quickly test these therapies.

Although not undisputed, it appears that dietary restrictions or supplements have a direct beneficial effect on the delay of GACR progression. Dietary measures do not have to go through extensive experiments (once the experimental models and biological read-outs are established) and do not need to go through lengthy clinical trials and FDA/EMA approval procedures. Repurposed medicines (FDA/EMA-approved in terms of toxicity for other indications) take at least 2–3 years; efficacy and mode of administration for GACR needs to be proven in experiments or in a clinical setting. RNA-based gene therapies through relevant oligo injections into the retina is a possibility to prevent short-term progression of GACR. However, these frequent and repeated ocular injections needed for this therapy may be traumatic for young children. Also, the field is struggling with efficacy issues: in controlled cell lines with certain defined severe mutations the therapy may be effective, but, recently, a number of clinical trials using RNA-based gene therapies have failed due to insufficient therapeutic effect. Development and application of this personalized therapy-per-mutation may take 5-10 years to reach market access. AAV replacement therapy prevents long term progression of the disease, but its development takes longer than the previously mentioned approaches. Pre-clinical and clinical trials may together take more than 10 years, as witnessed by the successful development of *RPE65* gene therapy for the genetic eye disease Leber Congenital Amaurosis and early-onset retinal dystrophy. Finally, future cell replacement therapy aimed at replacing already damaged cells, would be the only truly curative treatment, but is currently still in its research phase. Nonetheless, the first RPE cell replacement therapy for age-related macular degeneration, also relevant for patients with GACR, has recently been developed and has entered clinical trials last years (NCT04339764).

## State of the art of gene therapy of the liver for GACR: safety and efficacy

In a recent pre-clinical study, Boffa and coworkers demonstrated that liver-directed *OAT* gene replacement therapy in *OAT* deficient mice significantly reduced systemic plasma ornithine concentrations. This decrease in ornithine coincided with improved retinal structure and function as demonstrated by microscopy and ERG (Boffa et al, 2023). Indeed, these are striking results that show promise, and that are in line with the benefits of the consensus amino acid restriction therapy (Seker Yilmaz and Gissen, 2023). The liver gene therapy field has recently experienced notable successes and failures; for example: the liver directed gene therapy for a systemic disorder (hemophilia A; Valoctogene roxaparvovec) has been FDA-approved in 2023, with multiple other gene therapies in clinical trials. However, the field has also had setbacks: in 2022, after liver-directed gene therapy of more than 2000 patients with spinal muscular atrophy with gene therapy (onasemnogene abeparvovec-xioi), two treated children died of acute liver failure.

As Boffa and coworkers rightfully indicate themselves, there are a number of limitations with their approach. These include fundamental issues: are the results in mice directly translatable to humans? For GACR treatment, is the AAV5 or AAV8 vector previously used in (experimental) liver gene therapeutic studies also the best choice for to transfect the perivenous human hepatocytes where *OAT* is (normally) most highly expressed (Boon et al, 1999). Furthermore, what is the harm done when OAT is not only targeted to perivenous hepatocytes but also in part to periportal hepatocytes with the potential risk that ornithine is withdrawn from the urea cycle resulting in hyperammonemia? What are the potential liver or systemic toxicity risks? And, most importantly: how to avoid partial efficacy of AAV treatment early in life, because of liver size increase in the growing child (Baruteau et al, 2017). Next to the aforementioned fundamental issues, there are also a number of therapeutic questions. Boffa et al showed that early (6–10 weeks) AAV treatment in OAT mice models leads to a decrease in systemic and eye cup ornithine levels, while the dose-dependent treatment effect may take up to 12 months (Boffa et al, 2023), halfway the normal lifespan of a mouse. Although we do not know whether the results in mouse models can be translated one-to-one to human patients, this treatment time frame may not be effective for GACR patients, since severe choroidal retinal damage is already extensive in humans in the fourth decade of life.

Obviously, as gene therapy of the liver restores OAT in the liver, it will not normalize ornithine catabolism in the RPE and photoreceptors. It will only lower the availability of ornithine that will be supplied via the choroid. Indeed, this reasoning may be in line with our previous findings that protein metabolism in the RPE relating to sub-RPE drusen formation is fueled for approximately 70% from the systemic side, and 30% from local retinal sources (Bergen et al, 2019). Based on our previous research and clinical experience, lowering ornithine serum concentration alone is not enough to fully prevent retinal degeneration (Balfoort et al, 2021).

## Current state of the art of gene therapy of the eye in GACR: safety and efficacy

Compared to liver (or other organs), there are a number of reasons why the eye is particularly suitable for gene therapy. These have been extensively reviewed elsewhere (Hu et al, 2021). In summary: the eye is easily accessible and AAV vectors can be administered by local injections. There is already a vast experience with experimental and clinical (grade) AAV vector applications to successfully target specific cell types in the eye and retina. Indeed, for clinical applications affecting the RPE, the AAV2 vector has been successfully applied (Russell et al, 2017). Also, disease progression and therapeutic effect can be monitored non-invasively, with very high structural and functional detail. In case of potential toxic events, the blood-retina barriers separate the local environment of the eye from the rest of the system. The eye is therefore considered an immune-privileged site, and potential adverse effects of treatment will be confined to the eye. A large number of clinical ocular gene therapy trials are currently ongoing (NCT05407636; NCT05748873). One ocular gene therapy product, i.e. AAV-mediated gene replacement treatment for *RPE65*, has been fully developed successfully and is available on the market as the first-ever FDA-approved gene therapy (Russell et al, 2017).

## Conclusions

Taken together, there is reason for some optimism, as there are multiple (future) options to treat GACR, that are currently being investigated. These treatments include dietary restrictions, intake of supplements, (repurposed) medicine, gene therapy and cell replacement therapy. Targeting the liver with gene therapy may reduce systemic ornithine levels that may have beneficial effects in line with the results of dietary restriction of ornithine intake. However, in our view, based on patient needs, ethical considerations, safety, efficacy, and current therapeutic state-of-the-art, local gene therapy of the eye is an attractive therapeutic approach, perhaps combined with a mild diet or protective supplements.
